# Novel cancer treatment paradigm targeting hypoxia-induced factor in conjunction with current therapies to overcome resistance

**DOI:** 10.1186/s13046-023-02724-y

**Published:** 2023-07-18

**Authors:** Ting-Wan Kao, Geng-Hao Bai, Tian-Li Wang, Ie-Ming Shih, Chi-Mu Chuang, Chun-Liang Lo, Meng-Chen Tsai, Li-Yun Chiu, Chu-Chien Lin, Yao-An Shen

**Affiliations:** 1 Department of Pathology, School of Medicine, College of Medicine, Taipei Medical University, Taipei, 110301 Taiwan; 2 Graduate Institute of Clinical Medicine, College of Medicine, Taipei Medical University, Taipei, 110301 Taiwan; 3 Department of Internal Medicine, National Taiwan University Hospital, College of Medicine, National Taiwan University, Taipei City, 100225 Taiwan; 4 Departments of Pathology, Oncology and Gynecology and Obstetrics, Johns Hopkins Medical Institutions, 1550 Orleans StreetRoom 306, Baltimore, MD CRB221231 USA; 5 Sidney Kimmel Comprehensive Cancer Center, Johns Hopkins University School of Medicine, Baltimore, MD USA; 6 Faculty of Medicine, School of Medicine, National Yang-Ming Chiao Tung University, Taipei, 112304 Taiwan; 7 Department of Obstetrics and Gynecology, Taipei Veterans General Hospital, Taipei, 112201 Taiwan; 8 Department of Midwifery and Women Health Care, National Taipei University of Nursing and Health Sciences, Taipei, 112303 Taiwan; 9 Department of Biomedical Engineering, National Yang-Ming Chiao Tung University, Taipei, 112304 Taiwan; 10 Medical Device Innovation and Translation Center, National Yang Ming Chiao Tung University, Taipei, 112304 Taiwan; 11 Department of General Medicine, Taipei Medical University Hospital, Taipei, 110301 Taiwan; 12 Department of General Medicine, Mackay Memorial Hospital, Taipei, 104217 Taiwan; 13 School of Medicine, College of Medicine, Taipei Medical University, Taipei City, 110301 Taiwan; 14 International Master/Ph.D. Program in Medicine, College of Medicine, Taipei Medical University, Taipei, 110301 Taiwan

**Keywords:** Hypoxia, HIF-1, HIF-2, Chemotherapy, Target therapy, Radiotherapy, Immunotherapy, Metabolic therapy, Therapeutic resistance, Combination therapy

## Abstract

Chemotherapy, radiotherapy, targeted therapy, and immunotherapy are established cancer treatment modalities that are widely used due to their demonstrated efficacy against tumors and favorable safety profiles or tolerability. Nevertheless, treatment resistance continues to be one of the most pressing unsolved conundrums in cancer treatment. Hypoxia-inducible factors (HIFs) are a family of transcription factors that regulate cellular responses to hypoxia by activating genes involved in various adaptations, including erythropoiesis, glucose metabolism, angiogenesis, cell proliferation, and apoptosis. Despite this critical function, overexpression of HIFs has been observed in numerous cancers, leading to resistance to therapy and disease progression. In recent years, much effort has been poured into developing innovative cancer treatments that target the HIF pathway. Combining HIF inhibitors with current cancer therapies to increase anti-tumor activity and diminish treatment resistance is one strategy for combating therapeutic resistance. This review focuses on how HIF inhibitors could be applied in conjunction with current cancer treatments, including those now being evaluated in clinical trials, to usher in a new era of cancer therapy.

## Background

Hypoxia is a frequent phenomenon in cancer growth and progression that is linked to adverse outcomes [1]. Due to rapid proliferation in an enlarging tumor mass and aberrant microvessel architecture, cells in solid tumors are prone to oxygen deficiency. As most solid tumors contain heterogeneously dispersed regions of chronic or acute hypoxia, hypoxia has been identified as one of the intrinsic features of solid tumors [2]. In cancer biology, hypoxia confers increased propensity of chemoresistance, radioresistance, disease relapse, and potentiates cancer metastasis [3]. This phenomenon is well established by evidence from as early as the 1950s, when Gray et al. first described the negative association between hypoxia and the success of radiotherapy [4].

The key players behind these unfavorable effects are the hypoxia-inducible factors (HIFs), a family of transcription factors that drive cellular adaptation to hypoxia. Under hypoxic conditions, HIFs have the ability to activate hundreds of genes responsible for genetic instability, cell proliferation, angiogenesis, metastasis, glucose metabolism, growth factor signaling, immunosuppression, and the development of therapeutic resistance [5, 6]. Consistently, overexpression of HIFs is associated with resistance to cancer treatment, metastasis, and poor prognosis in patients [7].

**Fig. 1:**
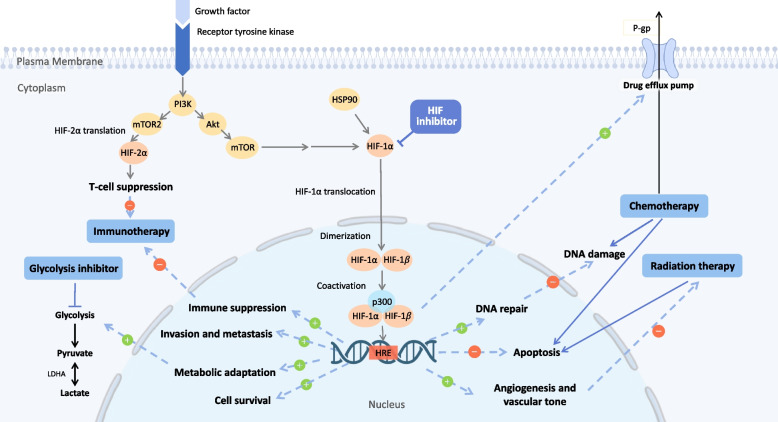
Targeting HIF in conjunction with current therapies. Resistance to chemotherapy, radiotherapy and immunotherapy is associated with the activation of HIF and its downstream. To improve anti-tumor effect and diminish treatment resistance, HIF inhibitors together with cancer therapies would be a good solution for patients with resistant cancer. Abbreviations: HIF = hypoxia inducible factor

Given the fact that cancer cells inhabit a unique hypoxic microenvironment that is often much more extreme than normal tissues and that hypoxia plays a major role in cancer progression and treatment resistance, it is only rational to utilize this characteristic to develop novel treatment modalities against cancer. Various strategies have been attempted to target hypoxia, including genetically engineered anerobic bacteria [8] and bioreductive prodrugs [9], which are activated in hypoxic environments. Among these approaches, the HIF pathway is a compelling target for pharmacologic inhibition due to its central role in hypoxic adaptative programs, versatility of effects, and selective induction under hypoxia. The correlation between HIF overexpression and poor treatment response has been demonstrated in an extensive range of cancers, including liver, ovarian, breast, cervical, pancreatic, colorectal, gastric cancer, and melanoma [6]. The co-administration of HIF inhibitors on top of current treatment modalities counteracts resistance and improves treatment response. This effect has been widely corroborated in chemotherapy [10], radiotherapy [11, 12], targeted therapy [13], and metabolic therapy [14], in various types of cancer, by preclinical studies and clinical trials. While numerous studies have demonstrated the efficacy of co-administering HIF inhibitors with existing cancer treatment modalities to overcome resistance and improve response rates, there remains a gap between the evidence in the literature and clinical practice, as current treatment guidelines do not yet incorporate this approach. Therefore, we herein propose that integrating HIF inhibitors into cancer treatment regimens represents a promising yet underutilized strategy for improving patient outcomes (Fig. 1).

Nevertheless, the development of HIF inhibitors has also faced various challenges and obstacles. Adverse drug-drug interactions and toxicity are major challenges when pursuing this line of research. This review summarizes the current progress of HIF inhibitor development and discusses the potential of combining HIF inhibitors with current treatment modalities as a novel cancer treatment approach.

## The functions and roles of HIFs in cancer biology

**Fig. 2:**
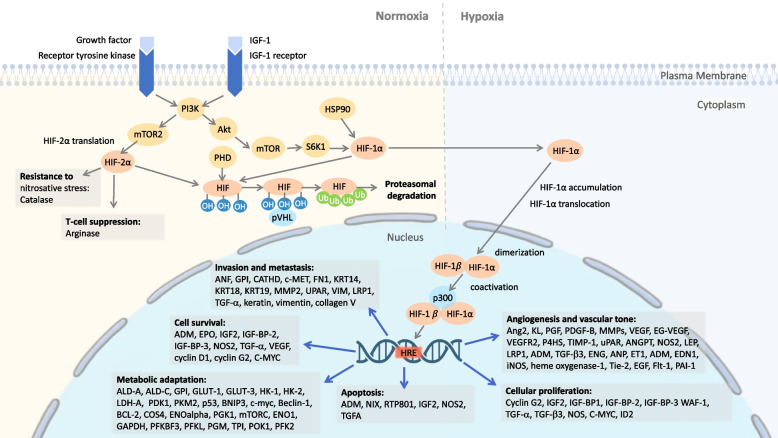
HIF-1a and HIF-2a upstream and downstream pathways promoting cancer. HIF-1a expression varies with oxygen level in the cellular environment while HIF-2a is constitutively expressed and has some known oncogenic effects. Under normal oxygen conditions, both HIF-1a and HIF-2a are degraded through ubiquitination pathway by proteasome. Under hypoxia (low oxygen) conditions, HIF-1a translocates into the nucleus and forms HIF complex with HIF-1b and p300, leading to many cancer-promoting outcomes, including metabolic adaptation, cell survival, invasion and metastasis, angiogenesis and vascular tone, cellular proliferation, and apoptosis. Abbreviations: ADM = adrenomedullin; ALD = adrenoleukodystrophy protein; ANF = atrial natriuretic factor; Ang2 = Angiopoietin 2; ANGPT = Angiopoietin-related protein; ANP = Atrial natriuretic peptide; BCL-2 = B-cell lymphoma 2; BNIP3 = BCL2/adenovirus E1B 19 kDa interacting protein 3; CATHD = Cathepsin D; c-MET = mesenchymal-epithelial transition factor; C-MYC = Cellular myelocytomatosis oncogene; COS4 = ; EDN1 = Endothelin 1 gene; EGF = Epidermal growth factor; EG-VEGF = endocrine gland derived vascular endothelial growth factor; ENG = Endoglin; ENO = Enolase; EPO = Erythropoietin; ET1 = Endothelin 1; Flt-1 = Fms Related Receptor Tyrosine Kinase 1; FN1 = soluble plasma fibronectin; GAPDH = Glyceraldehyde 3-phosphate dehydrogenase; GPI = Glycosylphosphatidylinositol; HIF = hypoxia inducible factor; HK = Hexokinase; HRE = Hypoxia-response element; HSP90 = Heat shock protein 90; ID2 = Inhibitor Of DNA binding 2; IGF2 = Insulin like growth factor 2; IGF-BP = Insulin-like growth factor-binding protein; iNOS = Inducible nitric oxide synthases; KL = Klotho; KRT = Keratin; LDH-A = Lactate Dehydrogenase A; LEP = Leptin; LRP = Low-density lipoprotein (LDL)-related protein; MMPs = Matrix metalloproteinases; mTORC = mechanistic target of rapamycin complex; NOS = nitric oxide synthases; OH = Hydroxyl group; P4HS = Prolyl 4-hydroxylase; PDGF-B = platelet derived growth factor; PDK1 = Pyruvate Dehydrogenase Kinase 1; PFK2 = Phosphofructokinase 2; PFKFB3 = 6-phosphofructo-2-kinase/fructose-2,6-bisphosphatase 3; PFKL = ATP-dependent 6-phosphofructokinase, liver type; PGF = Placenta growth factor; PGK1 = Phosphoglycerate Kinase 1; PGM = phosphoglucomutase; PHD = HIF prolyl hydroxylase; PKM2 = Pyruvate kinase isozymes M2; POK1 = Phragmoplast orienting kinesin 1; pVHL = Von Hippel–Lindau tumor suppressor; RTP801 = HIF responsive protein; S6K1 = Ribosomal protein S6 kinase beta-1; TGF = Transforming growth factor; TIMP = Tissue Inhibitor of Metalloproteinase; TPI = Triosephosphate isomerase; Ub = Ubiquitin; uPAR = urokinase plasminogen activator surface receptor; PAI-1 = plasminogen activator inhibitor 1; PI3K = Phosphoinositide-3-kinase; VEGF = Vascular endothelial growth factor; VEGFR2 = Vascular endothelial growth factor receptor 2; VIM = Vimentin; WAF-1 = Wild type p53 activated protein-1

HIFs are highly conserved transcriptional factors. The HIF-1 transcriptional complex is heterodimeric, composed of an alpha and a beta subunit, named HIF-1α and HIF-1β, respectively. Each subunit of HIF-1 consists of three isoforms, namely HIF-1α, HIF-2α, and HIF-3α, along with HIF-1β, HIF-2β, and HIF-3β. All are involved in cancer progression, HIF-1α regulates the response to acute hypoxia, while HIF-2α and HIF-3α are dominant in the chronic hypoxia response [2, 15]. In animal and clinical studies, HIF-1α and HIF-2α have been reported to participate in tumor growth, angiogenesis, and metastasis [15, 16] (Fig. 2). Although some inconsistent findings exist, most studies found that increased HIF-1α or HIF-2α expression is linked to a worse prognosis, and that HIF expression could be a potential biomarker for cancer therapy response [15, 17].

### Molecular functions of HIFs

Under normal oxygen conditions, HIF-1α subunit undergoes rapid proteasomal degradation due to hydroxylation by HIF prolyl-hydroxylases, allowing its recognition and labeling by the von Hippel-Lindau (VHL) E3 ubiquitin ligase [18]. Under hypoxia, HIF-1α is stabilized by its N-terminal transactivation domain and translocated into the nucleus, where it dimerizes with HIF-1β to form the HIF-1 complex [2]. The HIF-1 transcriptional complex subsequently activates a substantial number of cancer-related genes by binding to gene promoters containing hypoxia responsive elements (HRE) [1, 2, 15-17]. The HIF-1 regulated- genes include angiopoietin, vascular endothelial growth factor (VEGF), and erythropoietin, which promote angiogenesis and red blood cell production [1], glucose transporters (GLUTs) and glycolytic enzymes, which promote glycolysis [16], and genes involved in immune checkpoints [19], cell proliferation [20], autophagy [21], and DNA damage [22]. The discovery of HIF-1’s fundamental mechanism of oxygen sensing and adaptation was awarded the Nobel Prize in 2019 [23].

HIF-2α and HIF-3α are homologues of HIF-1α, bearing closely similar structures. Despite HIF-1α and HIF-2α sharing similar abilities to dimerize with HIF-β, bind to genes whose promoters containing the HRE motif, and activate downstream expression of hypoxia-inducible genes, they differ in expression levels during different timings in cancer progression [24]. Studies have shown that the expression of HIF-1α is more prominent in the early stages of cancer, during which tumorigenesis, cellular proliferation, and survival are major events, while HIF-2α-mediated cancer pathways are more active during the later stages, when metastasis and chemoresistance take place [25]. Various factors were suggested be involved in the signal switch from HIF-1α to HIF-2α, including prolonged hypoxia [26], pseudohypoxia [27], accumulation of Krebs cycle intermediates [28], onco-miRNAs [29], cytokines, and chemokines [30]. Upregulation of HIF-2α-mediated cancer pathways was reported to associate with advanced stage features such as malignant transformation, epithelial-mesenchymal transition, and VEGF resistance in a variety of cancers, especially gastric cancer [31], clear cell renal cell cancer (ccRCC) [32], and pancreatic cancer [33].

### Targeting HIFs in cancer

Because of the importance of HIF-1 and HIF-2 in cancer progression, substantial research has focused on developing compounds that suppress their effects [16, 34]. The inhibition of HIF presents a challenging target for pharmacological inhibition as transcription factors are intracellular protein complex without easily accessible active sites typically used for drug binding [35]. Until recently, most HIF inhibitors developed were indirect inhibitors that targeted different stages of HIF-1 pathway, such as mRNA expression, protein translation, degradation, DNA binding, and transcriptional activity. However, some small-molecule inhibitors have been identified that directly target the key protein–protein interactions involved in HIF-1 activation and downstream gene regulation [36]. Examples of indirect HIF inhibitors include EZN-2968, a synthetic antisense oligonucleotide that targets HIF-1α mRNA and leads to decreased HIF-1α protein expression [37], and PX-478, a prodrug that inhibits HIF-1α translation by blocking its interaction with ribosomes [38]. Direct HIF inhibitors, such as acriflavine and chetomin, offer exciting possibilities for drug discovery. Acriflavine is a small molecule dereived from natural compounds that disrupts the interaction between HIF-1α and its binding partner p300, preventing the transcriptional activation of downstream genes [39]. Chetomin, on the other hand, binds to the HIF-1α/ARNT heterodimer and inhibits its DNA binding activity [40]. Interestingly, many HIF inhibitors are repurposed drugs that were originally developed for other purposes, such as metformin, a widely used drug for diabetes that has been shown to inhibit HIF-1α expression [41]. This suggests that drug repurposing could also be a cost-effective and efficient way to identify new HIF inhibitors for cancer therapy.

Combination therapies that incorporate HIF-1 inhibitors with other cancer therapies have considerable potential to improve treatment efficacy, ameliorate side effects, and reduce drug doses [16]. In the next section, we reviewed current research on the synergistic effects of HIF-1 inhibitors with different categories of cancer therapy, such as chemotherapy, targeted therapy, radiotherapy, immunotherapy, and metabolic therapy.

## Chemotherapy or targeted therapy with HIF inhibitors

### Chemotherapy resistance

HIF-1 activation causes the transcription of multiple genes implicated in chemoresistance, such as genes augmenting DNA repair ability, blocking apoptosis, and promoting drug efflux and cellular metabolism [6, 42, 43]. For example, P-glycoprotein (P-gp), a membrane efflux pump encoded by the multidrug resistant protein 1 (MDR1) gene, is recognized as one of the major elements of chemoresistance for its ability to regulate intracellular drug concentration [44]. The upregulation of P-gp was attributed to HIF-1 expression and was linked to adverse clinical outcomes [45]. Consistently, HIF-1 inhibition was demonstrated to overcome chemoresistance by downregulating MDR1/P-gp expression in colon cancer cells [46]. Moreover, HIF-1-mediated signaling is also indicated in the transition of cancer cells from oxidative phosphorylation to glycolysis. Under hypoxia, the HIF-1-dependent expression of GLUT1 and carbonic anhydrase IX is increased. This metabolic reprogramming was recognized to favor cancer progression, treatment resistance, and immune evasion [47]. Similarly, a recent study proved that HIF-1α-induced glucose metabolic reprogramming contributes to 5-Fluorouracil (5-FU) resistance in colorectal carcinoma through activation of the PI3K/Akt pathway and upregulation of nuclear β-catenin [48]. Further studies demonstrated that HIF-1 pathways are associated with increased stem-cell like features, which confers an increased risk of clinical relapse following chemotherapy. HIF-1α was shown to synergize with Transforming growth factor-2 released by cancer-associated fibroblasts to promote GLI family zinc finger 2 expression in colorectal cancer stem cells (CSCs), resulting in enhanced stemness, dedifferentiation, and chemotherapy resistance [49]. This evidence highlights that targeting HIF-1 in cancer and cancer stem cells has a significant potential to efficiently counteract chemotherapy resistance [6, 43].

### Targeted therapy resistance

Mammalian target of rapamycin (mTOR) and VEGF inhibition are frequently adopted targeted therapy strategies that can yield considerable therapeutic efficacy. On the other hand, resistance to these therapies is attributed to changes in the tumor microenvironment that allow tumor growth by reducing reliance on VEGF [50]. It has been proven that HIF-1 and protein kinase B (AKT) are factors upstream of target blockage and are capable of driving tumor growth despite mTOR and VEGF inhibition [49]. HIF-1 neovascularization can also be initiated by cell-autonomous endothelial checkpoints independent of VEGF and become resistant to angiogenesis inhibitors [51]. Another mechanism of VEGF tyrosine kinase inhibitor resistance is through direct selection of cell subpopulations that can rapidly upregulate alternative proangiogenic pathways and have intrinsic hypoxia resistance [52]. Several studies of renal cell carcinoma and hepatocellular carcinoma treated with VEGF tyrosine kinase inhibitors sunitinib [53] and sorafenib [54] found that downregulation of HIF-1α and upregulation of HIF-2α with treatment can further stimulate the production of pro-angiogenic factors and cytokines to foster vascular endothelial cell proliferation.

In addition, hypoxia constitutes a significant mechanism that drives resistance to epidermal growth factor receptor (EGFR) inhibitors [55]. In non-small cell lung cancer, hypoxia promotes the expression of fibroblast growth factor receptor 1 (FGFR1), which, in turn, sustains EGFR signal through the MAPK pathway. This finding is substantiated by the restoration of sensitivity to EGFR inhibitors following the blockade of FGFR1 or MAPK by selective inhibitors [55]. Furthermore, another study reported that under hypoxic conditions, HIF-1α induces the expression and secretion of hepatocyte growth factor in pancreatic stellate cell, which in turn activates Met and the PI3K-AKT pathway, leading to resistance to EGFR inhibitor in pancreatic cancer [56]. Together, the above evidence suggests that HIF-mediated pathways contribute to an important part of targeted therapy resistance. By this token, inhibition of HIF pathways could potentially improve the efficacy of current targeted therapies.

### Synergistic effects of HIF inhibitors in different types of cancer

**Fig. 3:**
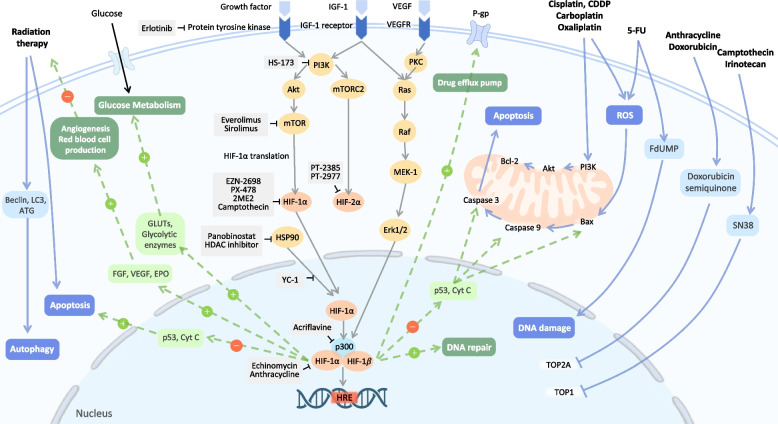
Inhibition of HIF-1α and HIF-2α in combination with chemotherapy, radiotherapy, and targeted therapy. Chemotherapy such as Cisplatin and 5-FU can cause DNA damage and cancer cell apoptosis. Radiotherapy can increase the level of ROS and lead to cancer cell apoptosis. However, during hypoxia, tumor cell HIF-1 pathway will be activated and result in therapy resistance. For chemotherapy resistance induced by HIF pathway, cancer cell can promote chemo-drug pump out, undergo DNA repairment, inhibit cell apoptosis and shift cell metabolism. And radiotherapy resistance is made by angiogenesis and more blood supply which promote cancer cell survival. Therefore, many synergistic effects by the combination therapy of HIF inhibitor and chemo, radio and targeted therapy through reversing the resistance by HIF pathway. The effects are seen in vivo and in vitro of varied cancer cell type. • Chemotherapy includes cisplatin, carboplatin, oxaliplatin, 5-FU, doxorubicin, irinotecan. • HIF-1 pathway inhibitor includes erlotinib (EGFR inhibitor); HS-173 (PI3K inhibitor); everolimus/sirolimus (m-TOR inhibitor); Panobinostat (HDAC inhibitor); EZN-2698, PX-478, 2ME2, Camptothecin (HIF-1α translation inhibitor); Acriflavine (HIF-1α dimerization inhibitor); Echinomycin and Anthracycline (HIF-1α DNA-binding inhibitor). • HIF-2 pathway inhibitor includes PT-2385 and PT-2977. • The impact of cancer therapy (White words with blue background). • The effect of activated HIF-1 pathway (White words with green background). If given HIF inhibitor, the downstream mechanism of HIF would decrease (Dotted lines). Abbreviations: PI3K = Phosphoinositide-3-kinase; Akt = protein kinase B; mTOR = mechanistic target of rapamycin VEGF = Vascular endothelial growth factor; VEGFR = Vascular endothelial growth factor receptor; EGF = Epidermal growth factor; IGF-1 = Insulin like growth factor-1; GLUT = Glucose transporter; BCL-2 = B-cell lymphoma 2; EPO = Erythropoietin; HIF = hypoxia inducible factor; HRE = Hypoxia-response element; HSP90 = Heat shock protein 90; ROS = Reactive oxygen species; TOP1 = Topoisomerase1

Chemotherapy combined with HIF inhibitors has a synergistic effect by reducing VEGF to constrain angiogenesis and endothelial proliferation, downregulating MDR1 expression to retain drug, inducing apoptosis, and redirecting glycolysis to oxidative phosphorylation, which leads to an increase in reactive oxygen species (ROS) production. The combined therapy in cell and xenograft models for different types of cancer was summarized in Table 1 and Fig. 3.

**Table 1:** Synergistic effect in different type of cancer via combined treatment of chemotherapy and HIF inhibitor

Cell	Cell line	Agent associated with HIF	Chemotherapy or Target therapy	Synergistic effects	Reference of study and 2D structure
**Brain**	Glioma	SN38: active metabolite of irinotecan		Decreases VEGF	PubChem CID: 104842 URL: https://pubchem.ncbi.nlm.nih.gov/compound/104842 [57]
	Glioma (D54MG)	HIF-1α knockdown	Temozolomide	Caspase activation for the cell death	PubChem CID: 5394 URL: https://pubchem.ncbi.nlm.nih.gov/compound/Temozolomide [58]
	Glioblastoma (U-87)	HIF-1α knockdown	Cisplatin	No synergistic effect	PubChem CID: 5702198 URL: https://pubchem.ncbi.nlm.nih.gov/compound/5702198 [59]
**Head and Neck**	Xenografts model (SQ20B)	Erlotinib: EGFR inhibitor	Cisplatin	Decreases VEGF and modulates tumor microenvironment	(E)PubChem CID: 176870 URL: https://pubchem.ncbi.nlm.nih.gov/compound/176870 [60]
**Lung**	Small cell lung cancer cells	NVP-ADW742: IGF-I receptor kinase inhibitor	Etoposide	Eliminates PI3K-Akt activity	(N)PubChem CID: 9,825149 URL: https://pubchem.ncbi.nlm.nih.gov/compound/9825149 (E)PubChem CID: 36462 URL: https://pubchem.ncbi.nlm.nih.gov/compound/Etoposide [61]
	Non-small cell lung cancer cells	Panobinostat: HDAC inhibitor	Cisplatin	Induces apoptosis, Activates caspases and PARP cleavage	(P)PubChem CID: 6918837 URL: https://pubchem.ncbi.nlm.nih.gov/compound/6918837 [62]
	Cells and xenograft model (A549)	Acriflavine		Prevents the formation of HIF-1α/β dimers	(A)PubChem CID: 443101 URL: https://pubchem.ncbi.nlm.nih.gov/compound/443101 [63]
	Cells and xenograft model (H460)	Oroxylin A		Binds to HIF-1α bHLH-PAS domain	(O)PubChem CID: 5320315 URL: https://pubchem.ncbi.nlm.nih.gov/compound/5320315 [64]
**Ovary**	Cells	NSC606985: Campothecin analog	Cisplatin	Antagonizes the accumulation of HIF-1α	(N)PubChem CID: 354677 URL: https://pubchem.ncbi.nlm.nih.gov/compound/354677 [65]
	Cells (OVCAR10 and SKOV-3)	RAD001 (everolimus)		Enhances cisplatin-induced apoptosis	(R)PubChem CID: 46930999 URL: https://pubchem.ncbi.nlm.nih.gov/compound/46930999 [66]
	Cells	Noscapine		Degrades cobalt-stabilized HIF-1α	(N)PubChem CID: 275196 URL: https://pubchem.ncbi.nlm.nih.gov/compound/275196 [67]
**Colorectal**	Cells	Acriflavine	Doxorubicin	Suppresses HIF-1 and the DNA replication	(D)PubChem CID: 31703 URL: https://pubchem.ncbi.nlm.nih.gov/compound/Doxorubicin [68]
	Cells (HT29)	L-carnosine	5-FU	Decreases multidrug resistant protein MDR1-pg	(L)PubChem CID: 439,224 URL: https://pubchem.ncbi.nlm.nih.gov/compound/439224 [5] PubChem CID 3385 URL: https://pubchem.ncbi.nlm.nih.gov/compound/5-Fluorouracil [69]
	Cells (SW1116)	Endostar	Oxaliplatin	Reduces HIF-2α and CXCR4 levels	(E)PubChem CID: 187888 URL: https://pubchem.ncbi.nlm.nih.gov/compound/187888 (O)PubChem CID: 43805 URL: https://pubchem.ncbi.nlm.nih.gov/compound/43805 [70]
	xenograft model	IDF-11774	5-FU	Restores 5-FU sensitivity in chemoresistant tumor	Pubchem CID: 71542096 URL: https://pubchem.ncbi.nlm.nih.gov/compound/71542096
**Liver**	Cells (Hep3B)	Melatonin	Sorafenib (S)PubChem CID: 216239 URL: https://pubchem.ncbi.nlm.nih.gov/compound/216239	Reduces HIF-1α mitophagy expression	(M)PubChem CID: 896 URL: https://pubchem.ncbi.nlm.nih.gov/compound/896 [71]
	Cells (HepG2)	2-Methoxyestradiol (2ME2)		Induces cell apoptosis Inhibits tumor angiogenesis	[2] PubChem CID: 66414 URL: https://pubchem.ncbi.nlm.nih.gov/compound/66414 [72]
	Cells	PT-2385: HIF-2α inhibitor		Suppresses HIF-2α Increases androgen receptor	(P)PubChem CID: 91754484 URL: https://pubchem.ncbi.nlm.nih.gov/compound/91754484 [73]
**Breast**	TNBC cells (Rb-positive)	Palbociclib: CDK4/6 inhibitor	BYL719: PI3K/mTOR inhibitors	Inhibits both CDK4/6/Rb/myc and PI3K/ mTOR signaling	(P)PubChem CID: 5330286 URL: https://pubchem.ncbi.nlm.nih.gov/compound/5330286 (B)PubChem CID: 56649450 URL: https://pubchem.ncbi.nlm.nih.gov/compound/56649450 [13]
			Paclitaxel	Downregulates the E2F target c-myc Reduces HIF-1α and GLUT1 expression	(P)PubChem CID: 36314 URL: https://pubchem.ncbi.nlm.nih.gov/compound/36314 [74]
	Cells and xenograft model (MCF-7)	Zoledronic acid (ZOL)	Fulvestrant	Inhibits ERK 1/2 phosphorylation	(Z)PubChem CID: 68740 URL: https://pubchem.ncbi.nlm.nih.gov/compound/68740 (F)PubChem CID: 104741 URL: https://pubchem.ncbi.nlm.nih.gov/compound/104741 [75]
	Xenograft model	CRLX101: TOPO-1 and HIF-1α inhibitor	Bevacizumab	Impedes the induction of both HIF-1α and cancer stem cell self-renewal	(C)PubChem CID: 184196 URL: https://pubchem.ncbi.nlm.nih.gov/compound/184196 [76]
**Pancreas**	Cells	PX-478	Gemcitabine (G)PubChem CID: 60750 URL: https://pubchem.ncbi.nlm.nih.gov/compound/60750	Elicits exposure of CRT Release of HMGB1 and ATP	(P)PubChem CID: 11234794 URL: https://pubchem.ncbi.nlm.nih.gov/compound/11234794 [77]
	Cells	FRAX597: PAK1 selective inhibitor		Inhibits HIF-1α and AKT activity	(F)PubChem CID: 70934541 URL: https://pubchem.ncbi.nlm.nih.gov/compound/70934541 [78]
	Cells (PANC-1)	HS-173: PI3K inhibitor	Sorafenib	Induces G2/M arrest Increases apoptosis	(H)PubChem CID: 52936849 URL: https://pubchem.ncbi.nlm.nih.gov/compound/hs-173 [79]

*Abbreviation*: *VEGF* Vascular endothelial growth factor, *EGFR* Epidermal growth factor, *PI3k* Phosphoinositide-3-kinase, *Akt* Protein kinase B, *IGF-1* Insulin like growth factor-1, *HDAC* Histone deacetylase, *MDR1* Multi-Drug Resistance 1, *GLUT1* Glucose transporter 1, *ERK ½* Extracellular-signal-regulated kinase ½, *m-TOR* Mechanistic target of rapamycin, *CDK* Cyclin-dependent kinases, *TOPO* Topoisomerase

SN38, a metabolite of irinotecan, which is both an HIF-1 inhibitor and a chemotherapy drug, was assessed in glioma cell models [57]. In another study, in comparison to monotherapy, temozolomide, an alkylating agent, can have a synergistic effect in the presence of HIF-1 knockdown [58]. There are no synergistic effects when HIF-1 knockdown glioblastoma cells are treated with cytotoxic chemotherapy; however, HIF-1 inhibition can led to oxygen-independent cytotoxicity and p53-independent apoptosis [59]. In another study, IDF-11774, a HIF-1α inhibitor, demonstrated a comparable ability to inhibit HIF-1α activity as HIF1A knock-down in vivo. Further investigation using a patient-derived xenograft murine model revealed that the combination of 5-FU and IDF-11774 successfully inhibited tumor growth in 5-FU-resistant rectal cancer. These results suggest that IDF-11774 has the potential to restore 5-FU sensitivity in treatment-resistant patients [48]. Erlotinib, an epidermal growth factor receptor inhibitor, can also suppress HIF-1 expression. A study showed that erlotinib combined with cisplatin can dampen tumor growth in a synergistic manner in head and neck squamous cancer cell and xenograft models [60].

NVP-ADW742 inhibits insulin-like growth factor-I-mediated VEGF and HIF-1α and can further boost sensitivity to etoposide and carboplatin in small cell lung cancer cell lines[61]. As for non-small cell lung cancer, acriflavine, which inhibits the development of HIF-1α/β dimers [63], panobinostat, which degrades HIF-1α and histone deacetylase 4 (HDAC4) [62], and oroxylin A, which binds directly to the HIF-1α bHLH-PAS domain [64], can reduce hypoxia-induced cisplatin resistance. Besides that, in ovarian cancer cells, cisplatin (CDDP) can better demonstrate its therapeutic potential when combined with NSC606985, a camptothecin analog that inhibits the accumulation of HIF-1α [65], noscapine, which results in the degradation of cobalt-stabilized HIF-1α [67], or RAD001 (everolimus), an mTOR inhibitor that suppresses the expression of HIF-1α [66]. Acriflavine, the most efficacious HIF-1 inhibitors [68], or l-carnosine, which reduces HIF-1α and MDR1-pg expression [69], can have synergistic effects with doxorubicin or 5-fluorouracil in colorectal cancer cells. Endostar, a customized recombinant human endostatin, can reduce HIF-2α and C-X-C motif chemokine receptor 4 (CXCR4) levels to overcome oxaliplatin-resistance [70]. Melatonin can inhibit the mTORC1/p70S6K/RP-S6 pathway to downregulate HIF-1α protein production and diminish cytoprotective HIF-1α-mitophagy expression in hepatocellular carcinoma, hence increasing sorafenib sensitivity [71]. Sorafenib blocks HIF-1α production, enabling the hypoxic response to shift from HIF-1α to HIF-2α-dependent pathways, allowing tumors to grow more aggressively [54]. 2-Methoxyestradiol can disturb the expression of HIF-1α and HIF-2α when combined with sorafenib [72]. PT-2385, a compelling HIF-2α inhibitor, can also improve sorafenib’s effectiveness and mitigate its undesirable side effects [73].

Palbociclib, a CDK4/6 inhibitor, can suppress HIF-1α and GLUT1 expression, and the combination with BYL719, BKM120, and BEZ235 (PI3K/mTOR inhibitors) can boost synergistic anti-proliferative and pro-apoptotic effects in breast cancer cells and a mouse model [13]. Additionally, the combination of palbociclib and paclitaxel can decrease cell growth more effectively than either therapy alone [74]. The combination of the HIF1 inhibitor zoledronic acid with fulvestrant decreases tumor development in vivo and in vitro [75]. In addition, the combination of CRLX101, a dual inhibitor of topoisomerase-1 and HIF-1, with bevacizumab results in enhanced tumor shrinkage and delayed tumor recurrence [76]. Under gemcitabine therapy, the addition of either PX-478, an HIF-1 inhibitor [77] or FRAX597, a PAK1 selective inhibitor [78] augments the antitumor efficacy and stifles pancreatic cancer cell proliferation and migration. Synergistically, the combination of HS-173 with sorafenib decreases cell viability, triggers G2/M arrest, and facilitates apoptosis with the loss of mitochondrial membrane potential [79].

As nanotechnology advances, there is growing interest in targeting the hypoxic tumor environment with nanoconstructs that exploit the controlled-release and site-specific properties of nanoparticles, as well as their capacity to serve as drug delivery platforms. A recent study proposed a calcium peroxide-modified magnetic nanoparticle (CaO_2_-MNPs) which releases oxygen at tumor sites and induces ubiquitin-mediated degradation of HIF-1α. In chemoresistant triple-negative breast cancer, combination of CaO_2_-MNPs with doxorubicin significantly inhibited tumor growth and induced apoptosis both in vitro and in orthotopic murine models [80]. On the other hand, another group of researchers coenstructed a chitosan-based nanoplatform that releases hematoporphyrin and oxygen-storing agent perfluorooctyl bromide which alters hypoxia tumor microenvironment. This drug delivery system demonstrated a synergistic effect with Erlotinib and sonodynamic treatment in three-dimensional tumor spheroids culture of non-small cell lung cancer cell lines through increasing the production of ROS and downregulating EGFR and HIF-1α [81].

## Radiotherapy with HIF inhibitors

The mechanism of radiotherapy to eradicate tumor cells is through either direct DNA damage or, more commonly, indirect damage by the induction of ROS [82]. Oxygen plays a crucial role in the success of radiotherapy since it affects the amount of ROS generated and the fixation of DNA damage via oxygen-free radical interaction [83]. In hypoxic conditions, radioresistance is increased due to reduced amounts of ROS and fixed DNA damage, which leads to a greater propensity for tumor cells to recover from DNA damage. Moreover, the reoxygenation of hypoxic tumor cells produces oxidative stress and leads to the upregulation of HIF-1, which stimulates the expression of VEGF and other pro-angiogenic factors that protect tumor cells from radiologic insults [84, 85]. Similarly, it has been demonstrated that the adaptive revascularization of tumors after radiation is dependent on the HIF-1-mediated pathway [86]. These studies suggest HIF-1 to be a major determinant of tumor survival after radiation and that disruption of the HIF-1 pathways is warranted in tumors that have developed radioresistance due to adaptive vascular recovery [87].

### Increased radiotherapy efficacy via HIF-1 pathway inhibition

 Numerous studies have examined the efficacy of radiotherapy alone vs. combined therapy (Fig. 3 and Table 2). For example, Erlotinib followed by radiation can decrease tumor regrowth [60]. Acriflavine can increase radiosensitivity and decompose endogenous H_2_O_2_ to O_2_. This HIF-1 inhibitor can further reduce hypoxia and boost ROS generation, resulting in DNA double-strand breakage and tumor cell apoptosis [88]. The diminished production of VEGF and matrix metalloproteinase 9 (MMP-9) further reduces the incidence of tumor dissemination [88]. Furthermore, in colorectal cancer cells, SN-38 may suppress HIF-1α and VEGF to cause cell cycle arrest in the S and G2/M phases, which elevates radiosensitivity [89]. Vitexin, a HIF-1 inhibitor, can increase the susceptibility of human glioma CSCs to hyperbaric oxygen when exposed to radiation [90]. Under hypoxic circumstances, the combined effect of sorafenib and radiation demonstrates synergistic cytotoxicity in breast CSCs by eliciting G2/M cell cycle arrest and inhibiting metastasis via reduction of HIF-1α and MMP-2 [91].

**Table 2:** Synergistic effect via combination of radiation and HIF inhibitor

Cell	Cell line	Agent associated with HIF and 2D structure	Mechanism	Reference of study and 2D structure
Colorectal cancer	HT29 and SW480	SN-38	Induces S and G2/M phases cell cycle arrest	PubChem CID: 104842 URL: https://pubchem.ncbi.nlm.nih.gov/compound/104842 [89]
Glioma	SU3	Vitexin: HIF-1α inhibitor	Reduces glutathione, glutathione peroxidase and GLUT1, GLUT3	PubChem CID: 5280441 URL: https://pubchem.ncbi.nlm.nih.gov/compound/5280441 [90]
Breast cancer stem cell	MDA-MB-231 and MCF-7	Sorafenib	Induces G2/M cell cycle arrest Inhibits metastasis	PubChem CID: 216239 URL: https://pubchem.ncbi.nlm.nih.gov/compound/216239 [91]
HNSCC	FaDu and ME180 xenograft model	HIF-1 knockdown	Increases oxygen consumption and killing of radiosensitive cell	[92]
	SQ20B	Rapamycin	Inhibits the mTOR/HIF-1α axis Elevation of HIF-2α	PubChem CID: 5284616 URL: https://pubchem.ncbi.nlm.nih.gov/compound/5284616 [93]
Non-small cell lung cancer	A549	Nelfinavir: HIV protease inhibitor	Decreases HIF-1α/VEGF expression	PubChem CID: 64143 URL: https://pubchem.ncbi.nlm.nih.gov/compound/64143 [94]
	A549	Endostar	Inhibits angiogenesis and tumor growth	PubChem CID: 187888 URL: https://pubchem.ncbi.nlm.nih.gov/compound/187888 [95]
	H460 and HCC2429	GSI: Notch pathway inhibitor	Inhibits Notch pathway and HIF-1	PubChem CID: 21741774 URL: https://pubchem.ncbi.nlm.nih.gov/compound/gamma-Secretase-Inhibitor-I PubChem CID: 5712 URL: https://pubchem.ncbi.nlm.nih.gov/compound/Lificiguat [96]
		YC-1: HIF-1 inhibitor		
Triple-negative breast cancer	MDA-MB-231	Chrysin	Inhibits HIF-1α and enhancing radiation-induced apoptosis	PubChem CID: 5281607 URL: https://pubchem.ncbi.nlm.nih.gov/compound/5281607 [97]

*Abbreviation*: *m-TOR* Mechanistic target of rapamycin, *GLUT1* Glucose transporter 1, *VEGF* Vascular endothelial growth factor

The majority of radiation combination treatment studies focused on squamous cell carcinoma of the head and neck (HNSCC) and non-small cell lung cancer cell lines. During transient hypoxic stress, HIF-1 knockdown in the xenograft model promotes hypoxia and boosts responsiveness to radiotherapy in HNSCC [92]. Cell lines treated with rapamycin and cetuximab can block the mTOR/HIF-1α axis. However, the increased expression of HIF-2α following the combination treatment increases the incidence of tumor recurrence [93]. Moreover, nelfinavir, an Human Immunodeficiency Virus protease inhibitor, reduces HIF-1α/VEGF expression in lung cancer cells, which can reverse radiation resistance [94]. When combined with radiation, endostar inhibits angiogenesis and tumor growth [95]. Another study proposed that upregulation of Notch pathway by radiation confers to radioresistance in non-small cell lung cancer cell lines through the activation of HIF-1α. The researchers showed that the radiation-induced upregulation of the Notch pathway and HIF-1α protein could be effectively suppressed by the HIF inhibitor YC-1, resulting in a synergistic antitumor effect when combined with radiotherapy under hypoxic conditions. Further combination of gamma-secretase inhibitor and YC-1 demonstrated the greatest radiosensitivity in vivo, suggesting a potential therapeutic strategy for future treatment [96]. Moreover, chrysin, a natural compound that has HIF1α and VEGF inhibiting capacity was also reported to act as a radiosensitizer in triple-negative breast cancer cell lines. When combined with radiotherapy, chrysin potentiates the effect of radiation by enhancing radiation-induced apoptosis and inhibiting STAT3 and cyclin D1 expression [97].

## Immunotherapy with HIF inhibitors

**Fig. 4:**
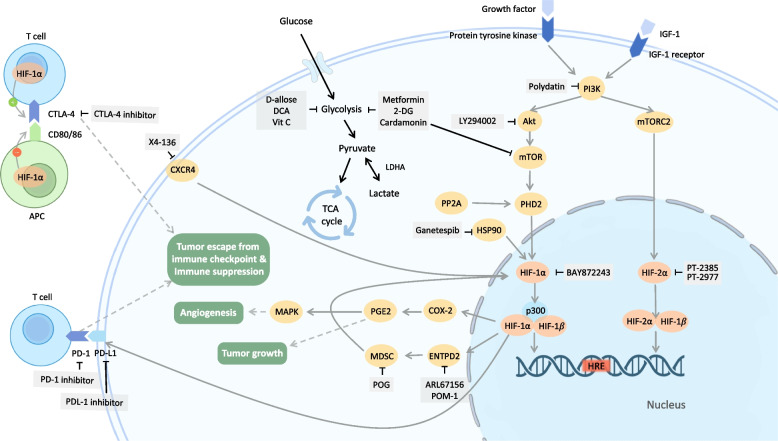
Combination therapy with immunotherapy or metabolic therapy. Combining blockage of HIF-1α or HIF-2α with immunotherapy (left). PD-1/PD-L1/CTLA-4, the immune checkpoint proteins on T cells which often transmit “immune switch off” signal, is correlated with HIF pathway, and tumor hypoxic status usually leads to the failure of immunotherapy. Therefore, by combining HIF/hypoxia inhibitors with PD-1/PD-L1/CTLA-4 inhibitors, it has been proved to significantly raise T cell immune activity, reducing the amount and malignant potential of tumors. • Immunotherapies: PD-1 inhibitor, PD-L1 inhibitor, CTLA-4 inhibitor. • HIF-1 pathway inhibitors: POG, X4-136, POM-1, ARL67156, Ganetespib. • HIF-2 pathway inhibitors: PT2385, PT2399. Combining blockage of HIF-1α or HIF-2α with metabolic therapy (right). Pyruvate flux through TCA cycle is downregulated in cancer cells. Glycolysis sustains the high proliferative rate of cancer cells. Targeting of HIF-1α may be a prerequisite for cancer metabolism targeted therapy. • Metabolic therapies: D-allose, dichloroacetate and vitamin C. • HIF-1 pathway inhibitors: polydatin, LY294002 and BAY872243. • Metabolic and HIF-1 pathway inhibitors: 2-DG, cardamonin and metformin. Abbreviation: IGF-1 = Insulin like growth factor-1; PI3K = Phosphoinositide-3-kinase; Akt = protein kinase B; mTOR = mechanistic target of rapamycin; mTORC2 = Mammalian Target of Rapamycin Complex 2; HIF = hypoxia inducible factor; HRE = Hypoxia-response element; PHD2 = prolyl hydroxylase domain protein 2; PP2A = Protein phosphatase 2A; HSP90 = Heat shock protein 90; CXCR4 = chemokine receptor type 4; COX2 = Cyclooxygenase-2; PGE2 = prostaglandin E2; MAPK = mitogen-activated protein kinase; ENTPD2 = Ectonucleoside Triphosphate Diphosphohydrolase 2; MDSC = Myeloid Derived Suppressor Cell; PD-1 = Programmed death-1; PD-L1 = Programmed death-ligand 1; CTLA-4 = cytotoxic T-lymphocyte-associated protein 4; CD80/86 = Cluster of differentiation 80/86; APC = Antigen-presenting cell; LDHA = Lactate dehydrogenase A; TCA = tricarboxylic acid

Even though the early responses of immune checkpoint blockade have been uplifting in various types of cancer, clinical data shows that most patients relapse in a short period of time, and the overall benefit of the therapy is far from fulfillment [98]. Hypoxic tumor microenvironment has been recognized as an attributor of the suboptimal results of immunotherapies [99, 100]. For instance, via HIF-1α and HIF-2α, hypoxia upregulates the expression of immune checkpoints to develop an immunosuppressive tumor microenvironment and facilitate tumor phagocytosis escape [98]. In the same vein, HIF-mediated induction of cancer cell glycolysis under hypoxia is a common contributor to immune checkpoint inhibitor resistance [101]. As anti-tumoral immune cells such as T cells and natural killer (NK) cells usually have a high glucose demand for their cytotoxicity, the glucose-deprived tumor microenvironment significantly compromises the effect of immunotherapies [101, 102]. Moreover, autophagy induced by hypoxia renders tumor cells less susceptible to NK cell-mediated cytotoxicity and cytotoxic T lymphocyte-mediated lysis [103]. According to HIF-1’s ability to bind HREs on HLA-G (human leukocyte antigen-G), hypoxia upregulates the expression of the non-classical and immunosuppressive major histocompatibility complex (MHC) Class I [104]. The above evidence infers that reducing hypoxia in tumors could attenuate immunosuppressive factors and block the activity of immunosuppressive cells while simultaneously increasing the number of cytotoxic T cells and lead to a general improvement of anticancer immunotherapy (Fig. 4). However, the combination of a HIF inhibitor and immunotherapy remains theoretical at this moment. Further preclinical and clinical studies are urgently needed to test the feasibility and effectiveness of combining HIF inhibitors with immunotherapy. The following section lists applications of HIF pathways in immunotherapies that could serve as the focus of combination therapy in future studies (Table 3).

**Table 3:** Synergistic effect via combination of immunotherapy and HIF inhibitor

Target	Immunotherapy agent	HIF inhibitor agent	Mechanism of HIF inhibitor	Cell	Cell line	Reference
PD-1	PD-1 Ab (clone RMP1–14, rat IgG2a)	PMN-MDSC inhibitor: POG	Inhibits HIF-1α expression	Melanoma	Mouse: B16F10	PubChem CID: 14034912 URL: https://pubchem.ncbi.nlm.nih.gov/compound/14034912 [105]
				Breast cancer	Mouse: 4T1	
	PD-1 Ab	IDF-11774	Inhibits HIF-1α expression	Prostate cancer	Mouse: RM-1	Pubchem CID: 71542096 URL: https://pubchem.ncbi.nlm.nih.gov/compound/71542096 [106]
PD-L1	PD‐L1 Ab (SP142)	HIF-2α antagonist: PT2385 PT2399	Both interfere the heterodimerization of HIF-2α with HIF-β	ccRCC	Mouse: XP164, XP373, XP453, XP454	PubChem CID: 91754484 URL: https://pubchem.ncbi.nlm.nih.gov/compound/91754484 PubChem CID: 91663289 URL: https://pubchem.ncbi.nlm.nih.gov/compound/91663289 [107-109]
CTLA-4	CTLA4 mAb (UC10-4F10; hamster IgG)	anti-RANKL mAb: denosumab	Downregulates HIF-1α protein expression	Melanoma	Mouse: B16F10, LWT1	[109] [110]
				Colon carcinoma	Mouse: CT26	
				Prostate carcinoma	Mouse:RM-1	
				Prostate adenocarcinoma	Mouse: Tramp-C1	
				Fibrosarcoma	Mouse: MCA1956	
PD-1, CTLA-4	PD-1 (clone RMP1-14) CTLA-4 mAb (clone 9D9)	ENTPD2 inhibitor: ARL67156 POM-1	Inhibit downstream product MDSC to upregulate HIF-1α	HCC	Human: MHCC97L PLC/PRF/5, Hep3B Mouse: Hepa1-6	PubChem CID: 146157453 URL: https://pubchem.ncbi.nlm.nih.gov/compound/146157453 PubChem CID: 71311259 URL: https://pubchem.ncbi.nlm.nih.gov/compound/71311259 [111]
PD-1, CTLA4	CTLA-4 Ab (9H10) PD-1 Ab (clone: 29 F.1A12, 135204)	Ganetespib	Inhibits Hsp90 and leads to HIF-1α degradation	Colon carcinoma	Mouse: MC38/gp 100	PubChem CID: 135564985 URL: https://pubchem.ncbi.nlm.nih.gov/compound/135564985 [112, 113] [114]
				Melanoma	Human: melanoma 2338, 2400, 2549, 2559, 2812	
PD-L1, PD-1, CTLA-4	Anti-PD-1 (clone RMP1-14)	CXCR4 antagonist: X4-136	Inhibits CXCR-4 to positively feedback HIF-1α	Melanoma	Mouse: B16-OVA	[115] [116]
	Anti-PD-L1 (clone: 10F.9G2)			Renal Cancer	Mouse: Renal Cancer Renca DM (Renca-2159-84H)	
	Anti-CTLA-4 Ab (clone: 9D9)					
IL-1	miR-144-3p mimic	COX2 inhibitor: celecoxib	Inhibits IL-1β/NF-κB/COX-2/ HIF-1α pathways	NSCLC	Human: A549	PubChem CID: 349,978,031 URL: https://pubchem.ncbi.nlm.nih.gov/substance/349978031 [117]

*Abbreviation*: *ccRCC* Clear cell renal cell carcinoma, *HCC* Hepatocellular carcinoma (HCC), *MDSC* Myeloid derived suppressor cells, *NSCLC* Non-small cell lung cancer, *PD-1* Programmed cell death protein 1, *PD-L1* Programmed death-ligand 1, *CTLA4* Cytotoxic T-lymphocyte-associated protein 4, *CXCR4* CXC-chemokine receptor 4, *COX2* Cyclooxygenase-2

### HIF-1α inhibitors improve immunotherapy efficacy

In human hepatocellular carcinoma (HCC), entonucleoside triphosphate diphosphohydrolase 2 (ENTPD2), a direct transcriptional target of HIF-1α, is predominantly upregulated in hypoxia [111]. It accelerates the development of HCC tumors and the formation of myeloid-derived suppressor cells (MDSC), allowing tumor cells to evade immune checkpoints and immunosuppressive activities [118]. Inhibition of ENTPD2 by ARL67156 and POM-1 reduces tumor growth and improves the effectiveness of immune checkpoint inhibitors of programmed death-1 (PD-1) (clone RMP1-14) and cytotoxic T-lymphocyte-associated protein-4 (CTLA-4) (clone 9D9), implying that ENTPD2 is a prospective cancer treatment target [111]. Similarly, the HIF-1α inhibitor IDF-11774 was found to enhance the efficacy of anti-PD-1 treatment in murine prostate cancer xenograft models [106]. Further analysis revealed that the combination of IDF-11774 with an anti-PD-1 antibody reduced the presence of immunosuppressive cells such as M2 macrophages and myeloid-derived suppressor cells, while increasing the number of effector T cells in the tumor microenvironment [106].

The expression of receptor activator of nuclear factor kappa B ligand (RANKL) is driven by HIF-1α and serves as a poor prognostic indicator in various types of malignancies [119-121]. It has been demonstrated that RANKL activation induces HIF-1 protein expression reciprocally [110]. There is evidence that RANKL inhibition with denosumab bolstered the effectiveness of the anti-CTLA4 monoclonal antibody (UC10-4F10) against solid tumors and metastases. The combination augments clusters of differentiation 8 (CD8), T-cell influx, and T-cell cytokine secretion, thereby inhibiting subcutaneous tumor growth [110].

Heat shock protein-90 (Hsp90) is a molecular chaperone involved in cell cycle regulation, hormone signaling, and cellular stress response [122]. HIF-1α is the downstream protein product of Hsp90; blocking Hsp90 will trigger the degradation of HIF-1α. Researchers have revealed that the Hsp90 inhibitor ganetespib effectively suppresses tumor growth in colorectal cancer, breast cancer, and esophageal cancer [123-125]. Furthermore, ganetespib has demonstrated a significant synergistic effect when used with immunotherapy agents for melanoma. The combination of ganetespib with anti-PD-1 (clone: 29 F.1A12, 135,204) and ganetespib with anti-CTLA4 (9H10) improved T-cell anti-tumor response and survival. Besides, a considerable increase in CD8 T-cells is found in the ganetespib and CTLA4 antibody (9H10) group [112, 113].

Celecoxib, a cyclooxygenase-2 (COX-2) inhibitor, has been demonstrated to have anti-tumor effects through impairing HIF-1α [126, 127]. Interleukin-1β (IL-1β) plays a vital role in the transformation between inflammation and cancer in lung adenocarcinoma via the IL-1β/NF-κB/COX-2/HIF-1α axis. The concurrent inhibition of IL-1β/miR-144-3p/WT1D by a miR-144-3p mimic and IL-1β/NF-κB/COX-2/HIF-1α by celecoxib suppresses lung adenocarcinoma cell growth with a synergistic effect [117].

Inflammatory signals initiate the recruitment of activated myeloid-derived suppressor cells (MDSCs) to tumor regions. MDSCs induce HIF-1α to express an immense amount of PD-L1 and ultimately suppress T cell activation [128]. Prim-O-glucosyl cimifugin (POG), a traditional Chinese medicine saposhnikovia root extract, impedes PMN-MDSC proliferation and metabolism by modulating arginine metabolism and the tricarboxylic acid cycle in order to target their immunosuppressive capabilities. Furthermore, when combined with immunotherapy, POG boosts the anticancer efficacy of the PD-1 inhibitor (clone RMP1–14) in mouse tumor models, intensifies CD8^+^ T-lymphocyte infiltration in tumors, and demonstrates a novel targeted approach for the PD-1 pathway [105].

CXCR4 was discovered to be overexpressed in a variety of cancer cells in response to hypoxia, hence contributing to cancer metastases. CXCR4 stimulates the accumulation of HIF-1α in the nucleus, hence urging the production of HIF-1α downstream genes. Nuclear HIF-1α increases CXCR4 transcription reciprocally to establish a positive feedback loop [116]. A growing body of evidence indicates that the CXCR4 inhibitor X4-136 inhibits the growth of murine B16/OVA melanoma tumors, diminishes immune-regulatory cell populations in the melanoma microenvironment, and also impairs the growth of renal cell carcinoma in Renca-DM syngeneic models [129]. Different levels of synergistic effects were observed when combining X4-136 with anti-PD-1, anti-PD-L1, or anti-CXCR4, including retardation of tumor growth, decline in regulatory T cells and infiltration of CD8^+^ cell, implying X4-136 is a pertinent agent to immune checkpoint inhibitors [129].

### HIF-2α inhibitors improve immunotherapy effectiveness

In ccRCC, the loss of the tumor suppressor VHL is a common oncogenic factor. Although both HIF-1α and HIF-2α can be activated by the loss of VHL, mounting evidence suggests that HIF-2α plays a more central role than HIF-1α in ccRCC [130]. Multiple studies suggest that, unlike in other cancers, HIF-2α is oncogenic while HIF-1α is tumor suppressive in ccRCC [131]. This concept is supported by the observation that HIF-2α expression is proportional to the degree of dysplasia, while HIF-1α levels are inversely related to dysplasia [132]. In vitro and animal models of ccRCC have also shown that HIF-2α blockage with small interfering RNA (siRNA) is sufficient to inhibit the transformation of VHL-/- RCC cells [133]. Moreover, several studies have indicated that PD-L1 expression in ccRCC is primarily regulated by the pVHL/ HIF-2α axis instead of HIF-1α [134]. The ability of this axis to induce PD-L1 expression in ccRCC highlights the potential of combining immunotherapy and HIF-2 inhibition. Ongoing clinical trials are evaluating this strategy by combining selective HIF-2α antagonist PT2385 with the anti-PD-1 nivolumab (NCT02293980), as well as assessing the combination of non-specific HIF inhibitors, such as Hsp90 inhibitor vorinostat (NCT02619253), with immunotherapy in early phase clinical trials.

## Metabolic therapy with HIF inhibitors

Most cancer cells produce energy by depending largely on glycolytic metabolism despite the presence of adequate oxygen. This unique metabolic reprogramming, referred to as the “Warburg effect,” not only provides essential cellular energy that sustains cancer cell growth but also generates a constellation of metabolic intermediates that are involved in the proliferation, adhesion, and invasion of cancer [135]. In addition to the Warburg effect, a hybrid metabolic state was observed in some aggressive cancer cell types, with coexisting glycolysis and oxidative phosphorylation (OXPHOS) that enhance the adaptation to environmental stress, invasiveness, and metastasis [136, 137]. With growing insight into cancer metabolism, metabolic therapy has become a promising therapeutic approach by targeting the distinctive energy metabolism, either upregulated glycolytic pathways or the hybrid phenotype, of cancer [137]. However, resistance to metabolic therapies has been reported due to upregulation of HIF-1 [138]. As a major regulator of tumor adaptation, HIF-1 promotes the expression of GLUT and glycolytic enzymes, thereby counteracting the effect of antiglycolytic agents. In support of this, a study demonstrated that the combination of dichloroacetate, a glycolysis inhibitor, and metformin, a mitochondrial electron transport chain inhibitor, synergistically increases cancer cell death in lung and breast cancer cell lines. However, this effect can be compromised by the up-regulation of the HIF-1α pathway, which induces the expression of glycolytic enzymes and leads to resistance to dichloroacetate/metformin therapy [139]. Moreover, HIF-1 activates the activity of pyruvate dehydrogenase kinase 1, which inhibits pyruvate dehydrogenase and suppresses the cell from undergoing mitochondrial metabolism [140]. These results challenge the utilization of metabolic therapy as a monotherapy for cancer and make targeting HIF-1α a prerequisite for cancer metabolic interventions. With this insight, increasing investigations have been looking into the combination of HIF-1 inhibitors and metabolic treatment, which may have a synergistic effect on suppressing cancer cell growth (Fig. 4). Although most studies are still in the preliminary stage, current preclinical results provide promising evidence for the potential of this novel treatment.

### Glycolysis inhibitors targeting the HIF pathway

HIF-1α protects mitochondria from the hexokinase 2 (HK2) inhibitor 3-bromopyruvate-induced damage. 3-bromopyruvate and the glucose analog 2-Deoxy-D-glucose (2-DG), when used together, have greater anticancer effects in pancreatic cancer cells, as they inhibit glycolysis as well as HIF-1α [141]. Using SB202190, a MAPK inhibitor, reduces the accumulation of HIF-1α protein. Pre-treatment with SB202190 renders the tumor more sensitive to PARP cleavage, which is mediated by the glucose analogs 2-DG and D-allose and induces apoptosis in pancreatic cancer cells [142]. Combining polydatin, a natural precursor of resveratrol, with 2-DG in MCF-7 and 4T1 breast cell lines can stimulate cancer cell apoptosis and restrict cell proliferation by inhibiting the ROS/PI3K/AKT/HIF-1α/ hexokinase 2 signaling axis [143]. Cardamonin inhibits glycolysis and the mTOR/p70S6K pathway by downregulating HIF-1α; this accelerates mitochondrial oxidative phosphorylation and ROS production, ultimately impeding the survival of the triple-negative breast cancer cell line MDA-MB-231 [144] (Table 4).

**Table 4:** Synergistic effect via combination of glycolysis inhibitor and HIF inhibitor

Drug	Agents associated with HIF-1	Mechanism	Cell line	Reference
3-BrPA 2-DG		Declines SOD1	MiaPaCa2 cell	[141] PubChem CID: 70684, 108223 URL: https://pubchem.ncbi.nlm.nih.gov/compound/Sodium-bromopyruvate https://pubchem.ncbi.nlm.nih.gov/compound/108223
2-DG D-allose	SB202190	Enhances PARP cleavage Inhibits LDHA gene expression	MIA PaCa-2, BxPC-3, ASPC-1, and SK-OV-3 cells	[142] PubChem CID: 108223, 439507, 5169 URL: https://pubchem.ncbi.nlm.nih.gov/compound/439507 https://pubchem.ncbi.nlm.nih.gov/compound/5169
Dichloroacetate (DCA)	LY294002	Blocks the Akt/GSK‑3β/HIF‑1α signaling	Pulmonary arterial smooth muscle cells (PASMCs)	[145] PubChem CID: 25975, 3973 URL: https://pubchem.ncbi.nlm.nih.gov/compound/25975 https://pubchem.ncbi.nlm.nih.gov/compound/3973
2-DG	LY294002 PD(Polydatin)	Inhibits ROS/PI3K/AKT/HIF‐1α/HK2	MCF-7 and 4T1	[143] PubChem CID: 5281718 URL: https://pubchem.ncbi.nlm.nih.gov/compound/5281718
Cardamonin		Represses the mTOR/p70S6K pathway	MDA-MB-231 cells	[144] PubChem CID: 641785 URL:https://pubchem.ncbi.nlm.nih.gov/compound/641785

*Abbreviation*: *SOD1* Superoxide dismutase 1, *PARP* Poly-ADP ribose polymerase, *LDHA* Lactate Dehydrogenase, *ROS* Reactive oxygen species, *m-TOR* Mechanistic target of rapamycin, *PI3k* Phosphoinositide-3-kinase, *Akt* Protein kinase B

### OXPHOS inhibitors that target the HIF pathway

Metformin, one of the OXPHOS inhibitors, dampens the transcription of the mitochondrial respiratory chain (complex I) and HIF-1α [146, 147]. Several investigations have demonstrated that combining metformin with chemotherapy or immunotherapy has synergistic effects in the treatment of cancer. Metformin may render oral squamous cell carcinoma (OSCC) cells more sensitive to cisplatin; the expressions of GLUT1 and BCL-2, which are target genes of HIF-1α, are decreased as a result of the inhibition of the NF-κB/HIF-1α signaling pathway [148]. Metformin modulates the AMPK/mTOR/HIF-1/P-gp and MRP1 pathways. It also has a synergistic anti-proliferative activity with 5-FU, which boosts apoptosis and provokes cell cycle arrest to reverse multidrug resistance in hepatocellular carcinoma [149]. Treatment of OSCC with metformin and 5-FU results in significant reductions in HIF-1α and mTOR and an increase in AMPKα [150]. Doxorubicin-metformin liposomes inhibit HIF-1α and tumor growth in multiresistant breast cancer cells, suggesting that this combination has the potential to revert HIF-1α-induced treatment resistance [151]. Furthermore, the effect of metformin combined with bevacizumab was also evaluated [152]. Although bevacizumab is typically ineffective in treating ovarian cancer, as it has the potential to worsen hypoxia in the tumor microenvironment, thus sustaining CSCs and promoting metastasis, a recent study showed promising results with the use of metformin in combination with bevacizumab. This combination, with or without cisplatin, successfully offset the increased HIF-1α and CSC population seen in bevacizumab monotherapy and suppressed the growth of ovarian cancer [152] (Table 5).

**Table 5:** Synergistic effect via combination of metformin and chemotherapy, target therapy, or immune therapy

Drug	Agents associated with HIF-1	Mechanism	Cell line	Reference
Vitamin C	BAY 87–2243	Inhibits the expression levels of VEGF via HIF‑1α‑dependent and AKT‑dependent pathways	Lens epithelial cells	[153] PubChem CID: 54670,067, 67377767 URL: https://pubchem.ncbi.nlm.nih.gov/compound/54670067 https://pubchem.ncbi.nlm.nih.gov/#query=67377767
Cisplatin	Metformin	Inhibits the NF-κB/ HIF-1α signal axis	Oral squamous cell carcinoma cells	[148, 154] PubChem CID: 5702198 URL: https://pubchem.ncbi.nlm.nih.gov/compound/5702198
5‑FU	Metformin	Targets the AMPK/mTOR/HIF‑1α/P‑gp and MRP1 pathways to reverse MDR	Hepatocellular carcinoma cells	[149] PubChem CID: 3385 URL: https://pubchem.ncbi.nlm.nih.gov/compound/3385
		Downregulates of HIF-1α and mTOR	Oral squamous cell carcinoma	[150]
Doxorubicin	Metformin	Inhibits HIF-1α and P-glycoprotein (Pgp) expression	Breast cancer cells (MCF7/ADR)	[151] PubChem CID: 31703 URL: https://pubchem.ncbi.nlm.nih.gov/compound/31703
Bevacizumab	Metformin	Inhibits VEGF, CD34, and HIF-1α expression	Ovarian cancer cells (SKOV3)	[152]
PD-1/ PD-1 antibodies (J43)	Metformin	Decreases tumor hypoxic status	Melanoma (Mouse: B16)	PubChem CID:4091 URL: https://pubchem.ncbi.nlm.nih.gov/compound/4091 [155]
			Colon carcinoma (Mouse:MC38)	
CTLA-4/ CTLA-4 mAb (Cat#BE0032;RRID:AB_1107798)	Metformin	Decreases tumor hypoxic status	Breast cancer (Mouse: 4T1-Luc2 Human: MDA-MB-231, MDA-MB-361, BT-549)	[156]
			Lung cancer (Human:H1975, H358)	
			Colon cancer (Human: RKO, CT26)	
			Melanoma (Mouse: B16-F10)	

*Abbreviation*: *VEGF* Vascular endothelial growth factor, *PI3k* Phosphoinositide-3-kinase, *Akt* Protein kinase B, *PD-1* Programmed cell death protein 1, *PD-L1* Programmed death-ligand 1, *CTLA4* Cytotoxic T-lymphocyte-associated protein 4

As for the combination of metformin with immunotherapy, it has been demonstrated that metformin not only reduces overall tumor hypoxia but also boosts antitumor cytotoxic T lymphocyte immunity by obstructing the PD-L1/PD-1 axis [155, 157]. The combination of metformin and PD-1 antibody (clone J43) elevates intra-tumoral T-cell activity and accelerates tumor clearance [155]. The combination of metformin with anti-CTLA-4 (clone 9D9) showed a substantial improvement in tumor shrinkage and cytotoxic T lymphocyte activity in 4T1 breast cancer, B16F10 melanoma, and CT26 colon cancer without significant body weight changes or observable damage in the kidneys or liver [156].

## Clinical trials

In addition to preclinical studies of HIF signaling pathways and combination effects with HIF inhibitors, several combinations have been employed in clinical trials during the past few years. There are several strategies for impeding HIF activity, including targeting HIF-1α protein synthesis, stability, dimerization, and interactions with other proteins [158]. For example, Everolimus (RAD001) is a kind of indirect HIF inhibitor that is frequently utilized in clinical studies [159]. Camptothecin and its analogs, including SN-38, topotecan, and irinotecan, are topoisomerase 1 inhibitors that also cause HIF-1α downregulation [160]. Moreover, Ganetespib, a Hsp90 inhibitor, has been shown to be capable of inhibiting HIF-1α activity [161]. HIF-2α inhibitors such as PT2385, PT2977 and DFF332 are further options [107]. Combination therapy with HIF inhibitors in clinical trials will be summarized in the following section (Table 6).

**Table 6:** The clinical trials studied the combination therapy with HIF inhibitors

HIF inhibitor	Combination agent	Study phase	Cancer	Status	Endpoint	Clinical trial number
Chemotherapy						
RAD001 (everolimus)	FOLFOX (oxaliplatin + 5 FU + leucovorin) Bevacizumab	Phase I/II	Metastatic CRC	Complete	MTD: 10 mg/d ORR: 53% PFS (6 mths): 96%	NCT01047293 [162]
Topotecan	Cisplatin Bevacizumab	Phase II	Cervical Cancer	Complete	PFS (6 mths): 59% mPFS: 7.1 mths mOS:13.2 mths	NCT00548418 [163] (T)PubChem CID: 60700 URL: https://pubchem.ncbi.nlm.nih.gov/compound/60700
Topotecan	Ziv-Aflibercept 5-Fluorouracil Folinic Acid	Phase II	Metastatic CRC	Complete	PFS (12 mths): 21.9% mPFS: 8.4 mths mOS: 20.9 mths	NCT02129257 [164]
Ganetespib (Hsp90 inhibitor)	Carboplatin Paclitaxel Radiation Therapy	Phase I	Stage II-III Esophageal Cancer	Complete	MTD (70 days)	NCT02389751
Ganetespib	Doxorubicin	Phase I/II	SCLC	Terminated	MTD: ganetespib 150 mg/m^2^ + doxorubicin 50 mg/m^2^ RR: 25%	NCT02261805 [165]
Ganetespib	Paclitaxel	Phase I/II	Metastatic, p53-mutant, Platinum-resistant Ovarian Cancer	Terminated	ORR: 20% (2 out of 10) DCR: 60% (4 out of 10) mPFS: 2.9 mths A: ganetespib 100 mg/m^2^ + paclitaxel B: ganetespib 150 mg/m^2^ + paclitaxel	NCT02012192 [166]
Targeted therapy						
SN-38	Cetuximab	Phase II	Metastatic or locally recurrent CRC	Complete	ORR: 10.7% PFS:4.9 mths	NCT00437268 [167] PubChem CID:104842 URL: https://pubchem.ncbi.nlm.nih.gov/compound/104842
Camptothecin	Bevacizumab	Phase II	Ovarian Cancer Fallopian Tube Cancer Primary Peritoneal Cancer	Complete	PFS (6 mths): 56% Camptothecin 15 mg/m^2^ + Avastin 10 mg/kg	NCT01652079 [168] PubChem CID: 184196 URL: https://pubchem.ncbi.nlm.nih.gov/compound/184196
RAD001	Sorafenib	Phase I/II	Advanced Solid Tumors	Suspended (toxicity)	MTD (6 wks) PFS (3mths)	NCT01226056
Ganetespib	Ziv-Aflibercept (VEGF-A and VEGF-B inhibitor)	Phase I	Metastatic gastrointestinal carcinomas, NSCLC, Urothelial carcinomas Sarcomas	Terminated (Drug supplier suspend)	MTD (28 days): ganetespib 100 mg/m^2^ + Ziv-Aflibercept 3 mg/kg	NCT02192541 [169]
Everolimus	Lenvatinib (RTK inhibitor)	Phase I	Advanced or metastatic RCC	Recruiting	Surgical complication	NCT03324373 (L)PubChem CID: 9823820 URL: https://pubchem.ncbi.nlm.nih.gov/compound/9823820
PT2977 PubChem CID: 117947097 URL: https://pubchem.ncbi.nlm.nih.gov/compound/117947097	Cabozantinib (TKI) PubChem CID: 25102847 URL: https://pubchem.ncbi.nlm.nih.gov/compound/25102847	Phase II	Advanced ccRCC	Complete	ORR: 22% DCR: 90% Median PFS: 16.8 months PFS rate(12mth): 65% OS rate (12mth): 81%	NCT03634540 [170]
PT2977	Abemaciclib (CDK4/6 inhibitors)	Phase I	Advanced refractory ccRCC	Recruiting	PR or CR MTD	NCT04627064 (A)PubChem CID: 46220502 URL: https://pubchem.ncbi.nlm.nih.gov/compound/46220502
Radiotherapy						
RAD001	Radiotherapy Erlotinib	Phase I	Recurrent head and neck cancer	Withdrawn	MTD	NCT01332279
Immunotherapy						
PT2385	Nivolumab	Phase I	Advanced or metastatic ccRCC	Active, not recruiting	MTD	NCT02293980 [171]
DFF332	Spartalizumab (anti-PD1) + Taminadenant (adenosine A2A receptor antagonist)	Phase I	Advanced or metastatic ccRCC	Recruiting	AEs DLT	NCT04895748
Metformin	Sintilimab (PD-1 inhibitor)	Phase II	ED-stage SCLC	Recruiting	ORR	NCT03994744 [172]

*Abbreviation*: *CRC* Colorectal carcinoma, *RCC* Renal cell carcinoma, *SCLC* Small cell lung cancer, *NSCLC* Non-small cell lung cancer, *PFS* Progression free survival, *mPFS* Median progression free survival, *OS* Overall survival, *DLT* Dose-Limiting toxicities, *MTD* Maximum tolerated dose, *AEs* Adverse events, *ORR* Objective response rate, *PR* Partial response, *CR* Complete response, *DCR* Disease control rate, *mths* Months, *yrs* Years, *NCT* number: https://clinicaltrials.gov/

### Everolimus

Everolimus, also known as RAD001, suppresses the accumulation and transcription of HIF-1α [173]. In a phase I/II clinical trial, RAD001 was administered with mFOLFOX-6 and bevacizumab for the treatment of metastatic colorectal cancer. In phase I, the maximum tolerated dosage (MTD) of RAD001 with mFOLFOX6 + bevacizumab was 10 mg daily. In phase II, 25 patients were daily administered 10 mg of everolimus. At MTD, the objective response rate was 53% and progression-free survival (PFS) was 96%. Accordingly, the combination of everolimus, mFOLFOX-6, and bevacizumab is tolerated and efficacious for the treatment of metastatic colorectal cancer [163]. Another ongoing phase I study (NCT03324373) is seeking to see if the combination of lenvatinib and RAD001 can render surgically unresectable metastatic renal cell carcinoma resectable. The primary objective was to examine surgical complications, and 15 patients were included. Another phase I/II study, which was supposed to investigate the use of sorafenib coupled with RAD001 in advanced solid tumors based on molecular targets, was suspended early due to toxicity (NCT01226056). The primary outcome of this phase I study was MTD, and progression-free survival at 6 months was for phase II. The other phase I trial (NCT01332279) investigated the optimal dose of RAD001, erlotinib, in conjunction with radiotherapy for the treatment of recurrent head and neck cancer. However, this research was discontinued due to a lack of funding.

### Camptothecin and analogues

Camptothecin, SN-38, topotecan, and irinotecan can lead to decreased accumulation and translation of HIF-1 because TOP1 plays a role as an upstream regulator in the HIF-1 pathway [160]. One phase II study investigated pegylated SN-38 or irinotecan plus cetuximab in patients with advanced colorectal cancer. The regimen was well tolerated for patients with refractory colorectal cancer, and overall survival and progression-free survival were similar in the cetuximab plus irinotecan arm and the SN-38 arm [167]. Another phase II trial evaluates the treatment effects of topotecan and cisplatin together with bevacizumab in recurrent or persistent cervical carcinoma. 6-month PFS was 59%, the median PFS was 7.1 months, and the median overall survival (OS) was 13.2 months. The results show the combination is an active but highly toxic regimen [163]. Another phase II trial investigates the FOLFIRI (leucovorin, 5-fluorouracil, irinotecan, and oxaliplatin) regimen combined with aflibercept in metastatic colorectal cancer patients. 73 patients were enrolled in the study, the median follow-up was 24.5 months, the 12-month PFS rate was 21.9%, median OS was 20.9 months, and the median PFS was 8.4 months. The combination regimen is tolerable; however, it did not improve the treatment efficacy in patients with metastatic colorectal carcinoma [164]. Another phase II trial (NCT01652079) investigated treatment of camptothecin and bevacizumab given in recurrent ovarian, tubal, and peritoneal cancer, the combination group received camptothecin 15 mg/m^2^ with Avastin 10 mg/kg which showed increased antitumor effect with PFS-6 56% compared to 27% in monotherapy group [168]. One phase I study (NCT00117013) is designed to explore the inhibition of HIF-1α expression and angiogenesis by topotecan in patients with metastatic solid tumors overexpressing HIF-1α. Topotecan was administered orally daily at a dose of 1.2 mg/m^2^ for 2 weeks, 16 patients were included in the study. Although the study was completed, the results are still pending.

### Ganetespib

A phase Ib/II trial evaluates the efficacy of the combination of ganetespib and doxorubicin in relapsed small cell lung cancer. Eleven patients were included: nine in the phase Ib dose escalation and two in the phase II expansion. The response rate was 25%, and the median duration of response was 137 days. The combination was well tolerated [165]. Another phase I trial studied ganetespib combined with Ziv-Aflibercept, a VEGF-A and VEGF-B inhibitor. Frequent grade 2 adverse events (50%) were observed with ganetespib 100 mg/m^2^ + Ziv-Aflibercept 4 mg/kg; however, with ganetespib 100 mg/m^2^ + Ziv-Aflibercept 3 mg/kg, decreased grade 2 adverse events were discovered. Thus, the MTD was not established yet owing to the toxicity [169]. The other phase I/II trial investigated ganetespib and paclitaxel in patients with platinum-resistant ovarian cancer, 10 patients were included: four patients were given paclitaxel and ganetespib 100 mg/m^2^, whereas paclitaxel and ganetespib 150 mg/m^2^ were given to six patients; no dose-Limiting toxicities was found in either group. Two patients achieved a partial response, and four patients had a 60% disease control rate. The median PFS was 2.9 months. The combination was tolerated without dose-Limiting toxicities [166]. Another phase I study investigated the side effects of ganetespib combined with paclitaxel, carboplatin, and radiotherapy in stage II-III esophageal cancer patients. The study enrolled three participants to determine the MTD, and it was completed but pending final results (NCT02389751).

### HIF-2α inhibitors

PT2385, a HIF-2α antagonist, can inhibit HIF-2α dimerization and its binding to DNA. In a phase I clinical trial (NCT02293980), PT2385 is given with nivolumab and cabozantinib to patients with metastatic renal cell carcinoma. The 50 patients were included in the expansion cohort of this clinical trial. The combination of PT2385 and nivolumab was tolerated, the objective response rate was 22%, and the median PFS of patients treated with PT2385 was 10.0 months [171]. PT2977, a second-generation HIF-2α inhibitor, has a similar mechanism to PT2385. However, PT2977 is more potent and has superior pharmacokinetics than PT2385. In a phase II clinical trial (NCT03634540) [170] investigating belzutifan plus cabozantinib for patients with advanced ccRCC, objective response rate was 22%, and disease control rate was 90%. The median PFS was 16.8 months, and the PFS rate and OS rate at 12 months were 65% and 81%, respectively. The study showed the combination had manageable safety and promising antitumor activity in patients with advanced ccRCC. Another phase I study (NCT04627064) aimed at assessing the safety and activity of abemaciclib and MK-6482 in patients with advanced refractory clear cell renal cell carcinoma. Moreover, another phase I study (NCT04895748) first used DFF332, a small molecule that targeted HIF-2α, in combination with Spartalizumab (anti-PD-1) plus Taminadenant (adenosine A2A receptor antagonist), in patients with advanced clear-cell renal cell carcinoma.

### Metformin

Metformin is not only a famous type 2 diabetes treatment to improve metabolic dysregulation but also a HIF-1α inhibitor to decrease hypoxia-induced HIF-1α accumulation [146, 147]. A phase II trial was launched in 2019 to study the efficacy and safety of the combination of the PD-1inhibitor sintilimab and metformin (NCT03994744) [172]. This trial included patients with extensive-stage small cell lung cancer who were resistant to or relapsed after standard chemotherapy. The trial is currently ongoing, and so far, no adverse events have been observed in the recruited cases.

## Future perspectives

In this review, we performed an extensive review of the literature on combination therapies involving HIF inhibitors. The synergistic impact of HIF-1α inhibitors with chemotherapy, targeted radiation, and immune therapy has been examined in cell lines and animal models with several promising results. In contrast, metabolic therapy has received less attention, and most results are still preliminary. Among the studied treatment combinations, some have proceeded to clinical trials, but they were mostly in phase I or phase II. Indirect HIF-1 inhibition is the most employed strategy. Recently, clinical trials on HIF-2α inhibitors such as PT-2385 and PT-2977 have been conducted; however, these trials were limited to clear cell renal carcinoma. Previous trials demonstrated a tolerable and effective outcome.

It is evident that the quantity and emphasis of preclinical and clinical trials investigating the synergistic impact of HIF inhibitors and cancer therapeutic drugs are escalating. However, several issues are to be addressed when perusing this line of research in the future. Firstly, since most currently adopted HIF inhibitors are repurposed drugs, their selectivity is often suboptimal. Off-target effects are major concerns of HIF inhibitors, but they could also be exploitable as inhibition of other HIF-mediated pathways may provide opportunities for additional antitumor effects. Second, in addition to evaluating synergistic effects in drug combinations, drug-drug interaction and toxicity are essential considerations when choosing HIF inhibitors. Third, HIF inhibitors-metabolic therapy combinations and applications of HIF-2 inhibitors are less investigated but are both potentially promising targets of research that are worth further attention. Lastly, more large-scale clinical trials are implicated in order to demonstrate the applicability of combined therapies in clinical settings.

## Conclusions

The HIF pathway plays a crucial role in the etiology of several malignancies. In addition to promoting cell proliferation and tumor invasion, HIFs are also essential for cancer therapeutic resistance. It has been proven that overexpression of HIFs contributes to poor treatment responses in an extensive range of malignancies. The addition of HIF inhibitors has the capacity to counteract resistance, and a synergistic effect was found in combination with chemotherapy, radiotherapy, targeted therapy, immune therapy, and metabolic therapy in preclinical studies. Several clinical trials have demonstrated that combination therapies are well tolerated and effective and that progression-free survival is improved relative to monotherapy. Overall, while further large-scale clinical trials are warranted, the addition of HIF inhibitors to cancer treatment regimens could become the new rationale for future cancer therapeutics.

## Data Availability

The data used to support the findings of this study are included within the article.

## Abbreviations

2-DG: 2-Deoxy-D-glucose
5-FU: 5-Fluorouracil
AKT: Protein kinase B
CaO_2_-MNPs: Calcium peroxide-modified magnetic nanoparticles
CSCs: Cancer stem cells
CAIX: Carbonic anhydrase IX
ccRCC: Clear cell renal cell cancer
CD: Cluster of differentiation
COX-2: Cyclooxygenase-2
CTLA-4: Cytotoxic T-lymphocyte-associated protein-4
CXCR4: C-X-C motif chemokine receptor 4
ENTPD2: Entonucleoside triphosphate diphosphohydrolase 2
EGFR: Epidermal growth factor receptor
FGFR1: Fibroblast growth factor receptor 1
GLUTs: Glucose transporters
GSI: Gamma-secretase inhibitor
HCC: Hepatocellular carcinoma
HDAC4: Histone deacetylase 4
HIFs: Hypoxia-inducible factors
HLA-G: Human leukocyte antigen-G
HNSCC: Squamous cell carcinoma of the head and neck
HRE: Hypoxia responsive element
Hsp90: Heat shock protein-90
ICB: Immune checkpoint blockade
IL-1β: Interleukin-1β
MDR1: Multidrug resistant protein 1
MDSC: Myeloid-derived suppressor cells
MHC: Major histocompatibility complex
MMP: Matrix metalloproteinase
MTD: Maximum tolerated dose
mTOR: Mammalian target of rapamycin
NK: Natural killer
OS: Overall survival
OSCC: Oral squamous cell carcinoma
OXPHOS: Glycolysis and oxidative phosphorylation
P-gp: P-glycoprotein
POG: Prim-O-glucosyl cimifugin
PD-1: Programmed death 1
PD-L1: Programmed death ligand 1
PFS: Progression-free survival
RANKL: Receptor activator of nuclear factor kappa B ligand
ROS: Reactive oxygen species
siRNA: Small interfering RNA
VEGF: Vascular endothelial growth factor
VHL: Von Hippel-Lindau
